# Extracellular vesicles as a liquid biopsy for amyotrophic lateral sclerosis: a systematic review and meta-analysis

**DOI:** 10.1186/s12967-026-08562-8

**Published:** 2026-07-15

**Authors:** Magdalena M. Bolsinger, Nandhana Vivek, Javraj Singh, Ashrit Challa, Amanda Zhu, Trent Rothell, Samuel Wang, Timothy Zhang, Shirley Zhu, Nathan Robbins, Leony Fenwick, Grant Ruttenberg, Aleksander Bogoniewski, Hash Brown Taha

**Affiliations:** 1 Friedrich-Alexander-Universität Erlangen-Nürnberg (FAU), Erlangen, Germany; 2 Department of Integrative Physiology, University of Colorado Boulder, Boulder, CO USA; 3 Washington University School of Medicine in St. Louis, St. Louis, MO USA; 4 Washington University in St. Louis, St. Louis, MO USA; 5 McMaster University, Hamilton, ON Canada; 6 University of California San Francisco, San Francisco, CA USA; 7 Department of Human Genetics, Radboud University Medical Center, Nijmegen, Netherlands; 8 Department of Molecular and Medical Pharmacology, University of California Los Angeles, Los Angeles, CA USA

## Abstract

Amyotrophic lateral sclerosis (ALS) is a fatal neurodegenerative syndrome diagnosed clinically using standardized criteria, with neuropathological confirmation of motor neuron loss and TDP-43 aggregates in postmortem brain tissue. Extracellular vesicles (EVs) have emerged as potential minimally invasive biomarkers for ALS, but studies vary widely in methodology and reproducibility. We conducted a systematic review and meta-analysis to evaluate the diagnostic potential of EV-associated proteins and RNAs in ALS. Following PRISMA guidelines, we searched PubMed and EMBASE from inception to May 21st, 2026. Forty-one studies met inclusion criteria. Where published summary statistics were available, these were used directly; where they were not, data were reconstructed from figures or obtained from authors and re-analyzed to derive standardized effect sizes and exploratory diagnostic accuracy estimates. Random-effects models were used for continuous outcomes, and diagnostic accuracy was assessed using hierarchical summary ROC and bivariate random-effects models. Publication bias was evaluated using Begg, Egger, and funnel plots. EV-associated TDP-43 was the most frequently studied protein. Meta-analysis of five studies showed a moderate but non-significant increase in EVs from ALS vs. controls (SMD = 1.30) with high heterogeneity (I = 97.8%). Sixteen studies assessing EV-RNA biomarkers showed minimal overlap and limited independent replication. Diagnostic accuracy meta-analysis across 11 studies yielded moderate performance (AUC = 0.839). No publication bias was found across both meta-analyses. EV biomarkers for ALS show biological promise but are limited by methodological variability and insufficient replication. This work highlights the need for standardized protocols, transparent data sharing, and independent validation.

**Supplementary information:**

The online version contains supplementary material available at 10.1186/s12967-026-08562-8.

## Introduction

Amyotrophic lateral sclerosis (ALS) is a fatal and complex neurodegenerative syndrome marked by the progressive degeneration of both upper and lower motor neurons. This neuronal loss leads to denervation of skeletal muscles and ultimately to paralysis. ALS is clinically diagnosed based on established clinical criteria [1]. Neuropathologically, cytoplasmic aggregation of TAR DNA-binding protein 43 (TDP-43) in degenerating motor neurons represents the defining hallmark of ALS, present in approximately 97% of persons with ALS [2, 3]. ALS caused by SOD1 mutations is typically characterized by the absence of TDP-43 proteinopathy [2, 3]. Although the precise etiology remains elusive, converging evidence indicates that ALS arises from a complex interplay of genetic susceptibility and environmental exposures [4]. While familial ALS with identified causative mutations only accounts for 5–10% of patients, most cases are sporadic [5].

ALS diagnosis relies primarily on clinical judgment, based on the recognition of characteristic motor features and exclusion of mimicking disorders. Despite refined consensus criteria, diagnostic confirmation remains challenging, particularly early in the disease course complicated by high clinical symptom heterogeneity. Consequently, delays between symptom onset and diagnosis are common, often extending over a year [6, 7]. Several interventions, including Riluzole, high‑caloric nutrition, and more recently the SOD1‑targeting antisense oligonucleotide tofersen (Qalsody), have demonstrated disease‑modifying effects in specific ALS subgroups, although the overall prognosis for most patients remains poor with a median survival following diagnosis of approximately three years [8–11]. Given diagnostic delays and the growing but still limited repertoire of disease‑modifying therapies, the development of validated, non‑invasive biomarkers is an urgent priority. Such biomarkers could enable earlier diagnosis, offer quantitative measures of disease progression (prognosis) and stratify patients for clinical trials to evaluate treatment outcomes (predictive). Numerous approaches have been explored to improve the diagnosis of ALS. These include neurofilament light chain (NfL) and phosphorylated NfL (pNfL) in cerebrospinal fluid and blood, neuroimaging techniques such as MRI and diffusion tensor imaging, electrophysiological testing, inflammatory and metabolic biomarkers, and genetic screening for common ALS-associated mutations [12–17], however, none of these modalities can definitively diagnose ALS.

Extracellular vesicles (EVs) are a heterogeneous population of small, membrane-bound vesicles, including ectosomes and exosomes [18]. Encapsulated by a phospholipid bilayer, EVs carry a diverse cargo of proteins, lipids, carbohydrates, and nucleic acids and function in intercellular communication and cellular waste disposal [19]. Notably, pathogenic proteins associated with ALS and other neurodegenerative diseases have been identified from EVs [20]. Because EVs may traverse the blood–brain barrier and reflect the molecular state of their cells of origin, both general EVs and so-called speculative CNS-enriched EVs have been proposed as minimally invasive biomarker sources for neurological and neurodegenerative disorders [21–24]. In our recent large-scale meta-analyses of parkinsonian disorders [22, 23] and dementia [24], we found that biomarkers derived from general EVs demonstrated superior diagnostic accuracy compared with speculative CNS-enriched EVs, with RNA-based EV biomarkers showing the strongest overall performance.

ALS-specific EVs for biomarker development and preclinical diagnosis have been investigated in recent years [25]. However, with differences in cohorts, biofluids, EV extraction methods, and analytical platforms, individual studies often yield variable and underpowered results. To allow clinical translation of identified biomarkers in basic research, a comprehensive understanding of the to date identified EV-associated biomarkers is needed. As such, we sought to systematically synthesize ALS‑related EV studies with explicit attention to methodological heterogeneity. In addition to qualitative synthesis, we performed targeted random‑effects meta‑analyses and exploratory diagnostic accuracy re‑analyses where data permitted, while transparently acknowledging the limitations imposed by small sample sizes, heterogeneous EV isolation methods, biospecimens, cargo types, and analytic platforms.

## Methods

We conducted a systematic review and meta-analysis following the guidelines of the Preferred Reporting Items for Systematic Reviews and Meta-Analyses (PRISMA). The study used only anonymized, previously published data and did not involve the collection of personal information or any direct research on human participants; therefore, ethical approval was not required. The study protocol was not registered as PROSPERO currently accepts only reviews for interventional studies.

### Data sources and search strategy

We performed a thorough search for relevant articles by using specific search terms for ALS and related disorders. The search was conducted in two databases (PUBMED and EMBASE) and covered articles published from the inception of the databases until May 21st, 2026. Three independent researchers (HBT, NV & JS) screened all the titles, abstracts, and full manuscripts to select articles that met the eligibility criteria. By hand, we reviewed the reference lists of eligible studies and searched Google scholar for articles using EVs as a liquid biopsy for ALS. Any discrepancies in article selection were resolved through discussion. The complete search strategy can be found in Table S1.

### Eligibility criteria

To be included in the systematic review (qualitative) eligible studies had to investigate EVs as a liquid biopsy approach for ALS using human-derived samples. We excluded studies that used animals or in vitro models (cell culture lines, primary cells and iPSCs) and studies that did not include the specified diseases. For studies that included longitudinal measurements or treatment interventions, we only considered the baseline assessments. Additionally, we contacted all authors to obtain other relevant information (see Table 1 for detailed information).

**Table 1:**
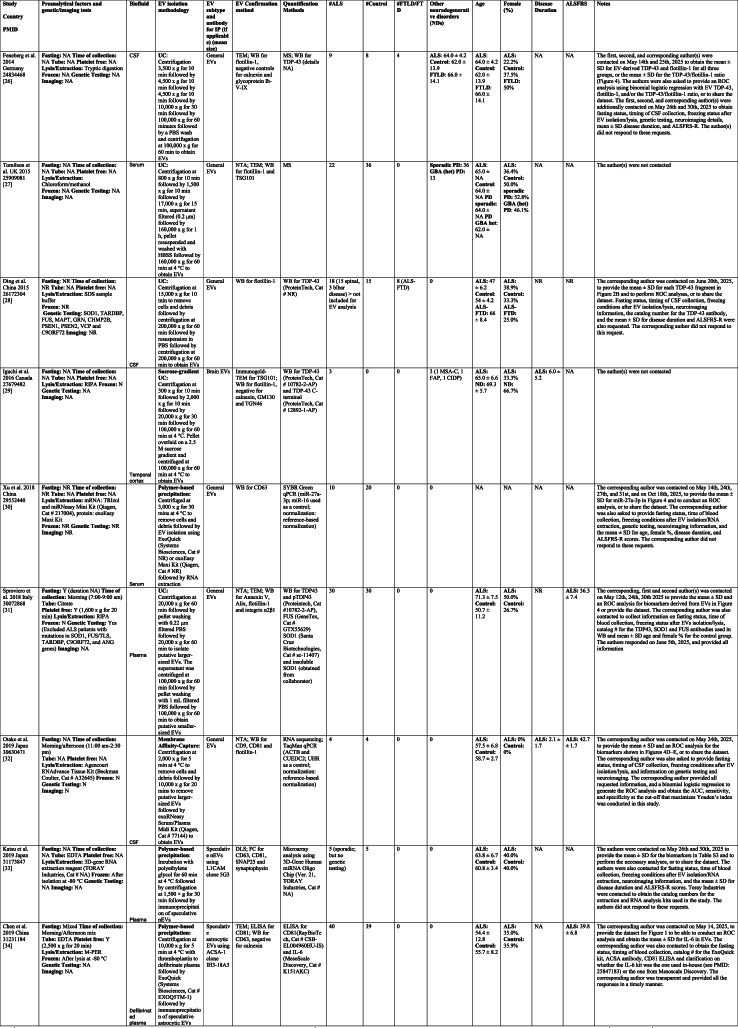

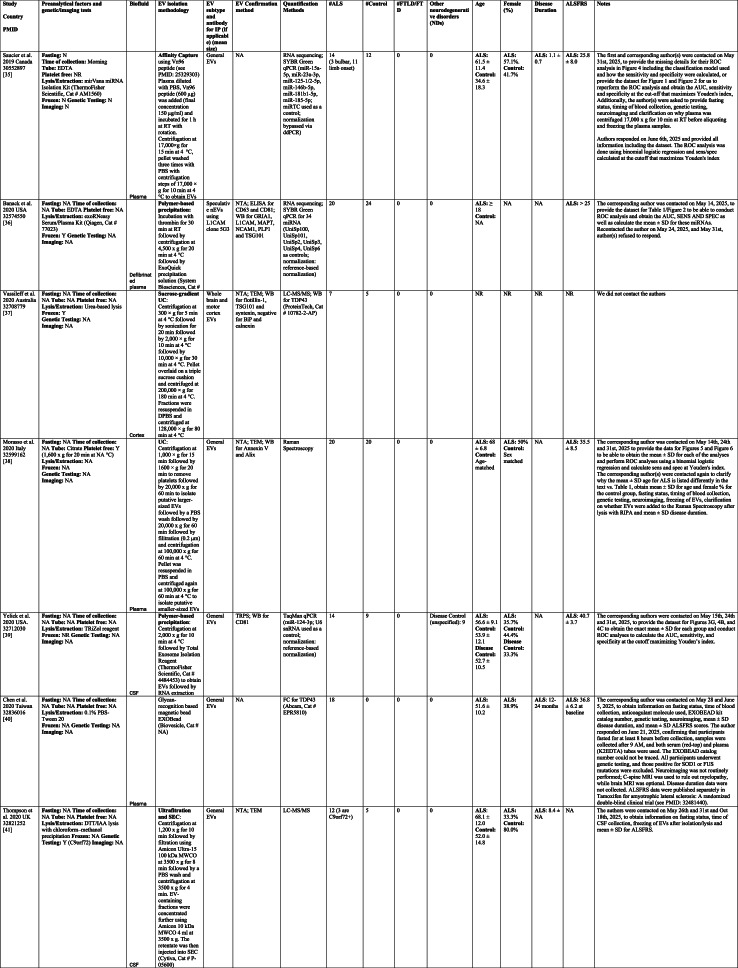

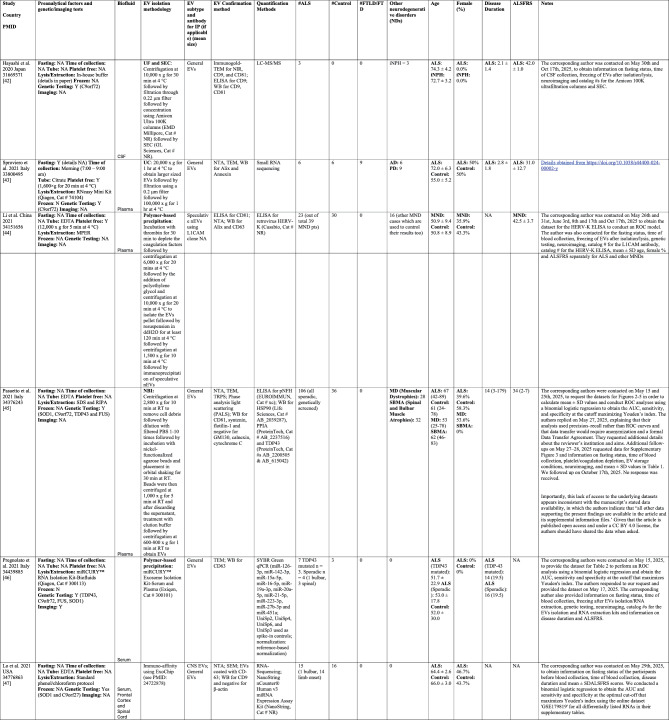

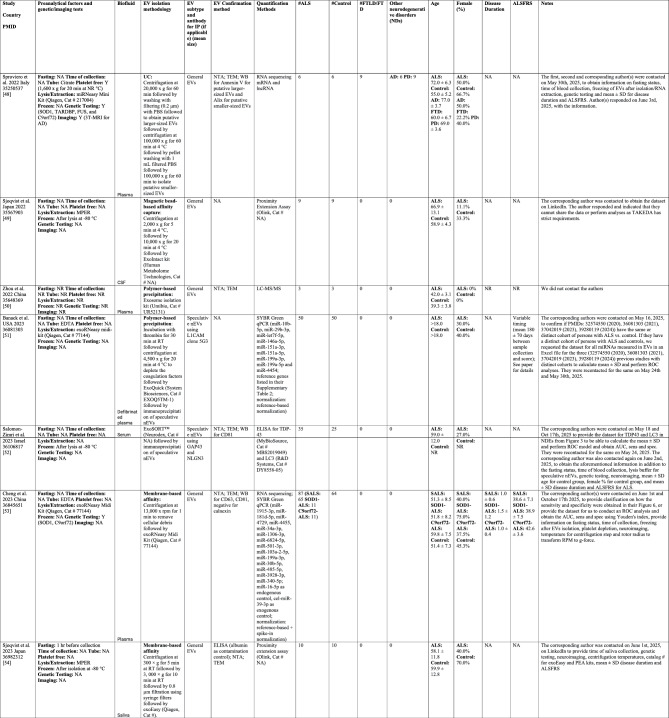

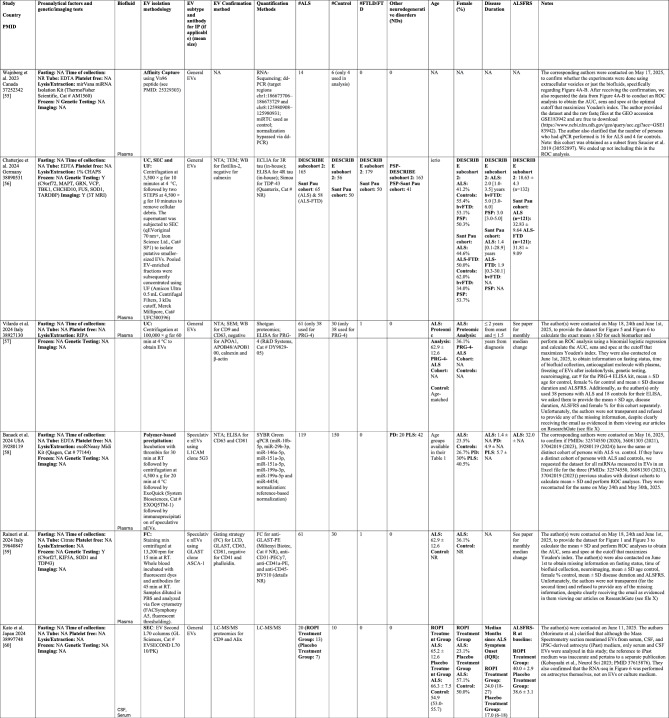

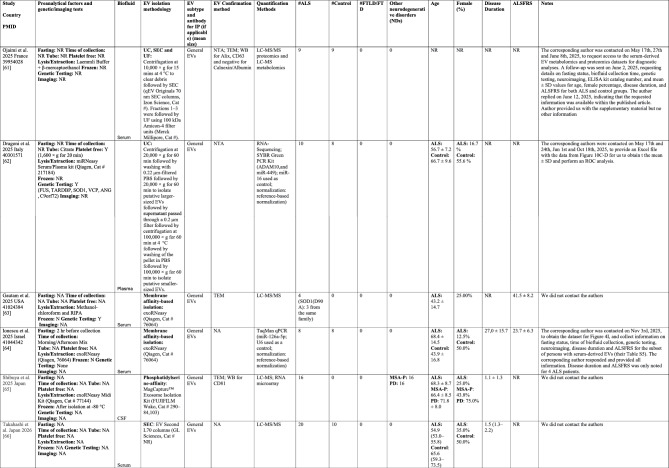
Demographics and characteristics of persons with ALS, controls, frontotemporal dementia (FTD) and other neurodegenerative disorders, along with extracellular vesicle (EV) isolation, confirmation and quantification methodologies used in studies included in the systematic review

**Abbreviations:** a Median (range). b Mean ± standard deviation (SD). c Mean ± standard error of the mean (SEM). d NR – not reported. e NA – not available/applicable. ALS – amyotrophic lateral sclerosis. SALS – sporadic amyotrophic lateral sclerosis. FTLD – frontotemporal lobar degeneration. FTD – frontotemporal dementia. MND – motor neuron disease. AD – Alzheimer’s disease. PD – Parkinson’s disease. PLS – primary lateral sclerosis. PSP – progressive supranuclear palsy. PSP-RS – progressive supranuclear palsy rating scale. iNPH – idiopathic normal-pressure hydrocephalus. MD – muscular dystrophy. SBMA – spinal and bulbar muscular atrophy. bvFTD – behavioural-variant frontotemporal dementia. ND – neurodegenerative disease. HC – healthy controls. CSF – cerebrospinal fluid. EVs – extracellular vesicles. nEVs – neuron-derived extracellular vesicles. NDEs – neuron-derived extracellular vesicles. SEVs – small extracellular vesicles. LEVs – large extracellular vesicles. UC – ultracentrifugation. UF – ultrafiltration. SEC – size-exclusion chromatography. NBI – nickel-based isolation. IP – immunoprecipitation. NTA – nanoparticle tracking analysis. DLS – dynamic light scattering. TRPS – tunable resistive pulse sensing. TEM – transmission electron microscopy. SEM – scanning electron microscopy. WB – Western blot. FC – flow cytometry. MS – mass spectrometry. LC–MS/MS – liquid chromatography–tandem mass spectrometry. RNA-seq – RNA sequencing. qPCR – quantitative polymerase chain reaction. dd-PCR – droplet digital polymerase chain reaction. PEA – proximity extension assay. ELISA – enzyme-linked immunosorbent assay. ECLIA – electrochemiluminescence immunoassay. RT – room temperature. PBS – phosphate-buffered saline. DPBS – Dulbecco’s phosphate-buffered saline. RIPA – radioimmunoprecipitation assay. CHAPS − 3-[(3-cholamidopropyl) dimethylammonio]-1-propanesulfonate. M-PER/MPER – mammalian protein extraction reagent. ALSFRS – Amyotrophic Lateral Sclerosis Functional Rating Scale. ALSFRS-R – Amyotrophic Lateral Sclerosis Functional Rating Scale–Revised. MMSE – Mini-Mental State Examination. MoCA – Montreal Cognitive Assessment. CDR-SB – Clinical Dementia Rating–Sum of Boxes. UPDRS III – Unified Parkinson’s Disease Rating Scale, part III. FAB – Frontal Assessment Battery. GLAST – glutamate aspartate transporter. Y – yes. N – no

To be included in the quantitative random-effects meta-analysis, studies were required to report a biomarker with mean and standard deviation (SD), along with the corresponding group sizes. Only baseline assessments were included to minimize confounding from disease progression, treatment effects, and other longitudinal changes that could influence biomarker measurements. For studies that did not provide mean and standard deviation, we searched for publicly available datasets and directly contacted the authors to request the summary statistics. If no dataset was available, we examined the figures to determine whether individual data points were overlaid; when present, we extracted those values using WebPlotDigitizer v3.4 (https://automeris.io/) to obtain the individual values and calculate the mean and SD. From one study [56], we estimated the mean and SD by transforming the median (Q1 + median + Q3)/(3) and IQR (Q3–Q1)/(1.35) as established previously [67].

To be included in the quantitative diagnostic accuracy meta-analysis, studies needed to have conducted an ROC analysis and reported the AUC, sensitivity, and specificity. Alternatively, studies were included if they provided the underlying dataset or included figures with overlaid individual values from which we could estimate these metrics using WebPlotDigitizer v3.4. We employed this framework to conduct a binomial logistic regression and obtain the sensitivity and specificity at the cutoff that optimizes Youden’s index [68].

### Data extraction

Data from eligible studies were extracted by at least two independent researchers. Authors checked the database for accuracy and completeness. Authors from the respective studies were contacted to obtain any missing information listed in Table 1. For quantitative outcomes, means and SD were taken directly from the original articles. When numerical values were not reported but individual data points were shown graphically, missing data were obtained with the open-source tool WebPlotDigitizer (https://automeris.io/). To verify the accuracy of the extraction, the digitized values were subsequently replotted in GraphPad Prism (version 8.0) and visually compared with the original figures. The close visual correspondence between the reconstructed and original plots supported the reliability of the extracted data. Based on the digitized values, summary statistics, including the mean and SD, were recalculated.

### Risk of bias assessment

Risk of bias was assessed using a modified version of the Newcastle–Ottawa Scale for cross-sectional studies [69]. Because all included studies evaluated EV biomarkers in a diagnostic context, studies were assessed based on how EV-associated proteins or miRNAs were measured cross-sectionally to compare individuals with ALS against controls or other clinically relevant comparator groups. Therefore, risk of bias assessment focused on the selection of study groups, comparability between groups, and ascertainment of EV biomarker exposure/outcome measures within this diagnostic framework. Risk of bias assessment was conducted by TZ and further checked by HBT.

### Data synthesis and statistics

Random-effects meta-analyses were performed in R software (version 2024.12.0 + 467) using the *metafor* package. An inverse variance weighting model was applied, and pooled standardized mean differences (SMD) with 95% confidence intervals were calculated based on Cohen’s d using the following formula:

$$d = {{{{\bar X}_1} - {{\bar X}_2}} \over {S{D_{{\rm{pooled}}}}}}$$

$$S{D_{{\rm{pooled}}}} = \sqrt {{{\left( {{n_1} - 1} \right)S_1^2 + \left( {{n_2} - 1} \right)S_2^2} \over {{n_1} + {n_2} - 2}}} $$

We performed diagnostic accuracy meta-analyses using the hierarchical summary ROC (HSROC) model or the bivariate random effects meta-analysis (BRMA) model, which are mathematically equivalent when no covariates are included [70]. These models were conducted using the *metadta* [71] command in Stata, which has been extensively validated in comparison to other packages [72]. Model selection (structured vs. unstructured covariance) was based on the lowest Akaike and Bayesian information criteria (AIC, BIC), with AIC used to break ties. Accuracy was interpreted from HSROC curves, where estimates closer to the upper left quadrant indicate higher performance. Heterogeneity was quantified using the I^2^ statistic of Zhou and Dendukuri [73].

Publication bias [74] was evaluated using Begg’s rank correlation, Egger’s and Deek’s regression tests and funnel plots. Studies with sensitivity and specificity = 1 were excluded from the diagnostic accuracy publication bias assessment. All publication bias analyses were performed in R software (version 2024.12.0 + 467).

Studies were included regardless of biofluid source (serum, plasma, defibrinated plasma) and EV isolation or quantification method. This decision was made to maximize the number of available studies; however, it introduces methodological heterogeneity that likely contributed to the high I^2^ observed. Furthermore, a relevant limitation is the abundant presence of TDP-43 in platelets, which can contaminate EV preparations. Only 1 of the 9 included studies analyzing TDP-43 levels reported specific platelet depletion or the use of platelet-poor plasma protocols, making it difficult to exclude platelet-derived TDP-43 as a potential source of signal.

### Terminology

We use the term general EVs to refer to EVs isolated using bulk isolation approaches that do not involve targeted immunoprecipitation for speculative CNS markers. The term speculative CNS-enriched EVs is used to refer to EVs enriched using speculative markers such as L1CAM for neuronal EVs (nEVs) or ACSA for astrocytic EVs, and CNS EVs to refer to EVs directly isolated from CNS tissue sources including brain and spinal cord. We use the term “speculative” in the before described context for at least three key reasons. The enrichment markers used are not specific to brain tissue and can also exist freely in circulation or on diverse peripheral cells. In addition, because EVs are continuously internalized and recycled by numerous cells throughout the body, any original signal they may have carried from the brain is likely diluted or altered. Lastly, no study has confirmed or proven that these EVs do indeed originate from the CNS.

## Results

### Cohort description

**Fig. 1:**
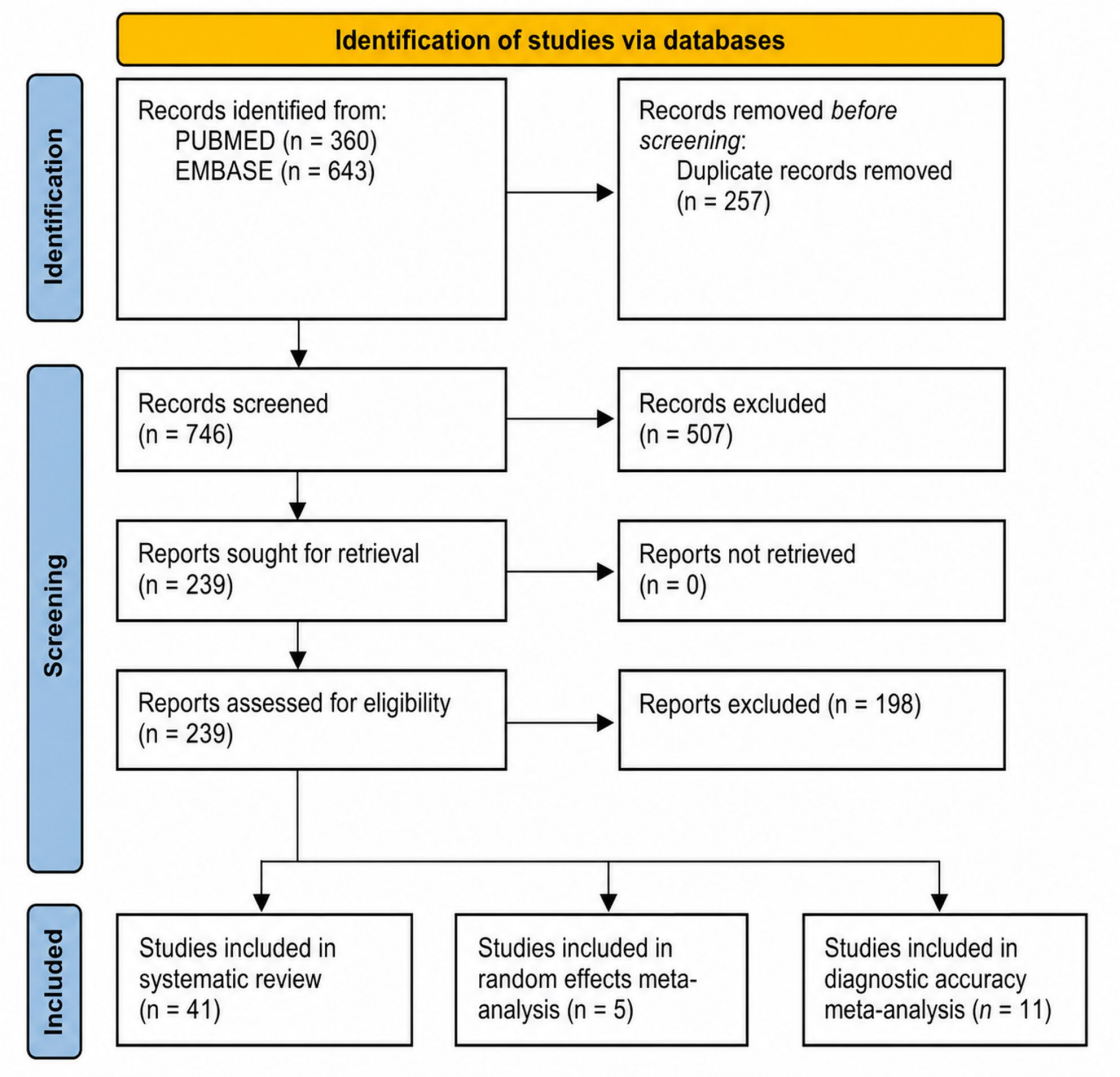
PRISMA flow diagram

Our systematic review encompassed 41 studies [26–66], of which 5 and 11 were included in the random effects and diagnostic accuracy meta-analyses, respectively (Fig. 1). The study population comprised 1,178 persons with ALS, 547 controls, 244 persons with frontotemporal dementia (FTD), 207 with progressive supranuclear palsy (PSP), 118 with other motor neuron diseases, 66 with ALS-FTD, 103 with Parkinson’s disease (PD), 12 with Alzheimer’s disease (AD), 3 with idiopathic normal pressure hydrocephalus, 19 with other amyloidogenic disorders, and 9 with unspecified diseases (see Table 1 for full details). Twenty-nine studies used general EVs. 7 studies used speculative CNS-enriched EVs using neural or glial markers (e.g., L1CAM, GLAST, ACSA-1, GAP43, NLGN3). One study used general EVs and speculative CNS-enriched EVs, 2 studies used CNS EVs, including whole brain, motor cortex and temporal cortex samples, and 1 study used general and CNS EVs from frontal cortex and spinal cord. Plasma was used in 17, CSF in 9, and serum in 10 studies. Defibrinated plasma was used in 3 studies, and saliva was used in 1 study. Among the studies, only 7 (23.3%) explicitly indicated platelet depletion in their protocols. In addition to peripheral biofluids, 3 studies examined CNS tissue sources, including temporal cortex, frontal cortex, motor cortex, and spinal cord. Across the included studies, ultracentrifugation (UC) was the most frequently used EV isolation method (11 studies), including sucrose gradient-UC (2 studies). Polymer-based precipitation was used in 10 studies, primarily through ExoQuick. Membrane-affinity based approaches such as exoRNeasy or exoEasy were applied in 5 studies, while combinations of size exclusion chromatography (SEC), ultrafiltration (UF), and UC were reported in 5 studies. Less frequently used approaches included affinity capture (Vn96 peptide) (2 studies), while EXOBead, nickel-based isolation, ExoSORT™, MagCapture™ and flow cytometry were reported in only 1 study each. In terms of EV characterization, 20 studies report TEM, 19 studies report NTA or comparable particle sizing/counting, and 22 studies include western blots (WBs) for EV markers (such as CD63, CD81, TSG101, syntenin or flotillin) and negative markers (such as calnexin, GM130 or mitochondrial/ER proteins), whereas the remaining 13 studies either provide minimal EV phenotyping or only indirect confirmation. The studies providing either minimal phenotyping or only indirect confirmation are indicated in Table 1. This limitation should be considered when interpreting their findings in the overall context of our study.

Protein-focused studies quantified specific cargo such as total or phosphorylated TDP‑43 (pTDP-43), C‑terminal TDP‑43 fragments, SOD1, FUS, HSP90, pNFH, HERV‑K, total tau, 3 R/4 R‑tau, IL‑6 and tetraspanins using WB, ELISA, single molecule array or mass spectrometry. Seventeen studies were RNA-centered and analyzed miRNAs, mRNAs, lncRNAs or unannotated RNAs, typically testing panels of 10 to 30 prespecified miRNAs, more than 80 RNAs in multiplex platforms, or discovering dozens to more than 100 dysregulated RNAs per EV fraction via small RNA‑seq, total RNA‑seq, microarrays, qPCR or ddPCR.

### Risk of bias assessment

Study methodological quality was assessed using modified Newcastle-Ottawa Scales adapted for cross-sectional studies. Overall, studies demonstrated fair methodological quality, with recurrent limitations related to sample representativeness, ascertainment of ALS exposure, pre-analytical handling, and adjustment for confounding variables. Across studies (Figure S1), 19 studies included sufficiently representative samples of individuals with ALS across varying severities and disease activity, while 36 studies selected controls from the same hospital population as exposed participants. Twenty-one studies had adequate ascertainment of exposure, with ALS diagnoses established using the El Escorial criteria or through post-mortem biopsies. Several studies attempted to account for the influence of age and sex on their findings, whereas adjustment or consideration of other clinically relevant variables, such as disease severity measured by ALSFRS was more limited. Thirty studies utilized targeted analytical methods, including qRT-PCR or flow cytometry, to measure EV biomarkers, and 29 studies confirmed EV isolation using 2 or more characterization techniques. Only 12 studies adequately described pre-analytical EV handling procedures, including factors such as fasting status, collection timing, collection tubes, platelet depletion, freezing conditions, or lysis methods.

## Proteins

### TDP-43, SOD1 and FUS

TDP-43, SOD1, and FUS are key proteins implicated in the pathogenesis of ALS with abnormal aggregation, mislocalization, or mutation of these proteins contributing to motor neuron dysfunction and degeneration [75]. TDP-43 pathology is present in the vast majority of ALS cases and is considered a defining neuropathological hallmark [75]. In our systematic review, 9 studies have quantified TDP-43 in general EVs isolated from CSF [26, 28], plasma [31, 40, 45, 56], serum speculative CNS-enriched EVs [52] and CNS-EVs [29, 37].

In CSF, 2 studies suggested that TDP-43 levels may be higher in those with FTD pathology as compared to ALS alone. The first study detected the presence of TDP-43 derived from general EVs of individuals with ALS using WB, but subsequent mass spectrometry analysis, with normalization to flotillin-1, did not show a statistically significant difference between ALS and controls, although TDP-43 trended toward higher levels in FTD [26]. In contrast, the second study [28] demonstrated that various TDP-43 species analyzed via WB (full-length TDP-43, TDP-35, and TDP-25) were higher in ALS-FTD vs. controls. Both studies suggest that CSF EV-associated TDP-43 and its cleaved products may be more prominently increased in ALS-FTD than in ALS alone.

A recent seminal study [56] with two independent large cohorts (named DESCRIBE SUBCOHORT and Sant Pau) measured TDP-43 in plasma general EVs using single molecule array. Across cohorts, plasma EV TDP-43 was consistently highest in ALS. Specifically, in the DESCRIBE subcohort, that was divided into a pilot (subcohort 1) and validation cohort (subcohort 2), TDP-43 levels in ALS were distinctly higher when compared to controls, behavioral variant FTD (bvFTD), and PSP. Furthermore, EV-associated TDP-43 distinguished ALS from controls, bvFTD, and PSP with AUCs around 0.91–0.99. Cases with confirmed TDP-43 pathology had markedly elevated EV TDP-43 compared to controls, PSP-type tau pathology, MAPT mutation carriers, and non-TDP-43/non-tau cases. Non-TDP-43/non-tau cases (SOD1, FUS, CHCHD10) had low TDP-43, similar to controls and PSP. In the second cohort of the aforementioned study (Sant Pau), the pattern was replicated: ALS and ALS-FTD had the highest plasma EV TDP-43 levels exceeding those of controls and PSP. However, in the Sant Pau cohort a subset of bvFTD cases also showed elevated EV TDP-43 levels, clustering with ALS. In the DESCRIBE subcohorts 1 and 2, EV TDP-43 outperformed plasma NfL, a widely used marker for neurodegeneration [76], in distinguishing bvFTD from controls and PSP, as well as ALS/ALS-FTD from controls and PSP in cohorts where NfL data were available. The same pattern was observed in the Sant Pau cohort where genetic cases associated with TDP-43 pathology (such as C9orf72, GRN, VCP, TBK1) showed high plasma EV TDP-43 levels, whereas genetically confirmed non-TDP-43/non-tau cases (SOD1, FUS) had low levels, comparable to controls and PSP. Using cohort-specific cut-offs for TDP-43 levels (for example > 13.87 or >17.85 pg/ml), EV TDP-43 levels accurately classified individuals' TDP-43 pathology with high sensitivity and high specificity in both cohorts and robustly detected sporadic ALS and ALS-FTD vs. controls and PSP. Additionally, EV plasma TDP-43 behaved like a severity marker correlating with disease severity and plasma NfL in both ALS and bvFTD.

These findings were previously supported by a study [40] of 18 people with ALS, where plasma EV TDP-43 ratios were measured at baseline and at 1-, 3-, 6-, and 12-month follow-up visits using flow cytometry. EV TDP-43 was significantly higher at 3 and 6 months compared with baseline, with a borderline increase at 12 months, suggesting progressive accumulation of EV-associated TDP-43 over time. Additionally, another study measured plasma EV TDP-43 levels in putative larger-sized and smaller-sized EVs using WB. Total TDP-43 and pTDP-43 were higher in putative larger-sized EVs from persons with ALS compared to controls, while no significant differences were observed in smaller-sized EVs [48]. Larger EVs were defined operationally by EVs derived from the pellet after 20,000×g centrifugation while smaller EVs corresponded to the 100,000×g pellet. Importantly, the ranges of TDP-43 levels in ALS and control samples overlapped substantially, indicating that although putative larger-sized EVs showed an overall increase in TDP-43, these levels alone were insufficient to reliably distinguish individuals with ALS from controls.

It is worth noting that one study reported that TDP-43 detection in general plasma EVs by WB was challenging, due in part to interference from abundant plasma proteins such as immunoglobulins and albumin when using antibodies against the N- or C-terminus. Although a pTDP-43 antibody produced a 45 kDa doublet in both ALS and control samples, immunogold TEM failed to confirm intravesicular localization, suggesting the signal was not truly EV-associated. This raises concerns about the specificity and reproducibility of measuring TDP-43 in plasma general EVs [45].

From another study [52] measuring TDP-43 in speculative CNS-enriched nEVs using ELISA, TDP-43 levels were higher in people with ALS compared to controls at baseline. In the longitudinal treatment arm (treated with a combination of ciprofloxacin and celecoxib), EV-associated TDP-43 levels decreased over time, while conventional plasma markers such as NfL and pNfH remained largely unchanged. In a postmortem study [74], CNS EVs isolated from the temporal cortex of 3 individuals with sporadic ALS contained significantly higher levels of TDP-43, including full-length and C-terminal fragments, compared with EVs isolated from individuals with other neurologic disorders (1 multiple system atrophy, 1 familial amyloid polyneuropathy, and 1 chronic inflammatory demyelinating polyneuropathy). However, this finding must be interpreted with considerable caution given the extremely small sample size (*n* = 3). In a postmortem study [29], general EVs isolated from the motor cortex of individuals with ALS were significantly enriched in the 28 kDa C-terminal fragment of TDP-43 compared with neurological controls, indicating that ALS brain-derived EVs carry higher levels of pathogenic TDP-43 species. This may support a direct link between CNS TDP-43 pathology packaging into EV cargo in ALS [37].

Studies measuring SOD1 and FUS1 in EVs are scarce; however, one study [31] reported that SOD1 and FUS1 were higher in plasma putative larger-sized EVs from persons with ALS compared to controls, whereas no clear disease-related differences were seen in putative smaller-sized EVs.

### TDP-43 random-effects meta-analysis

**Fig. 2:**
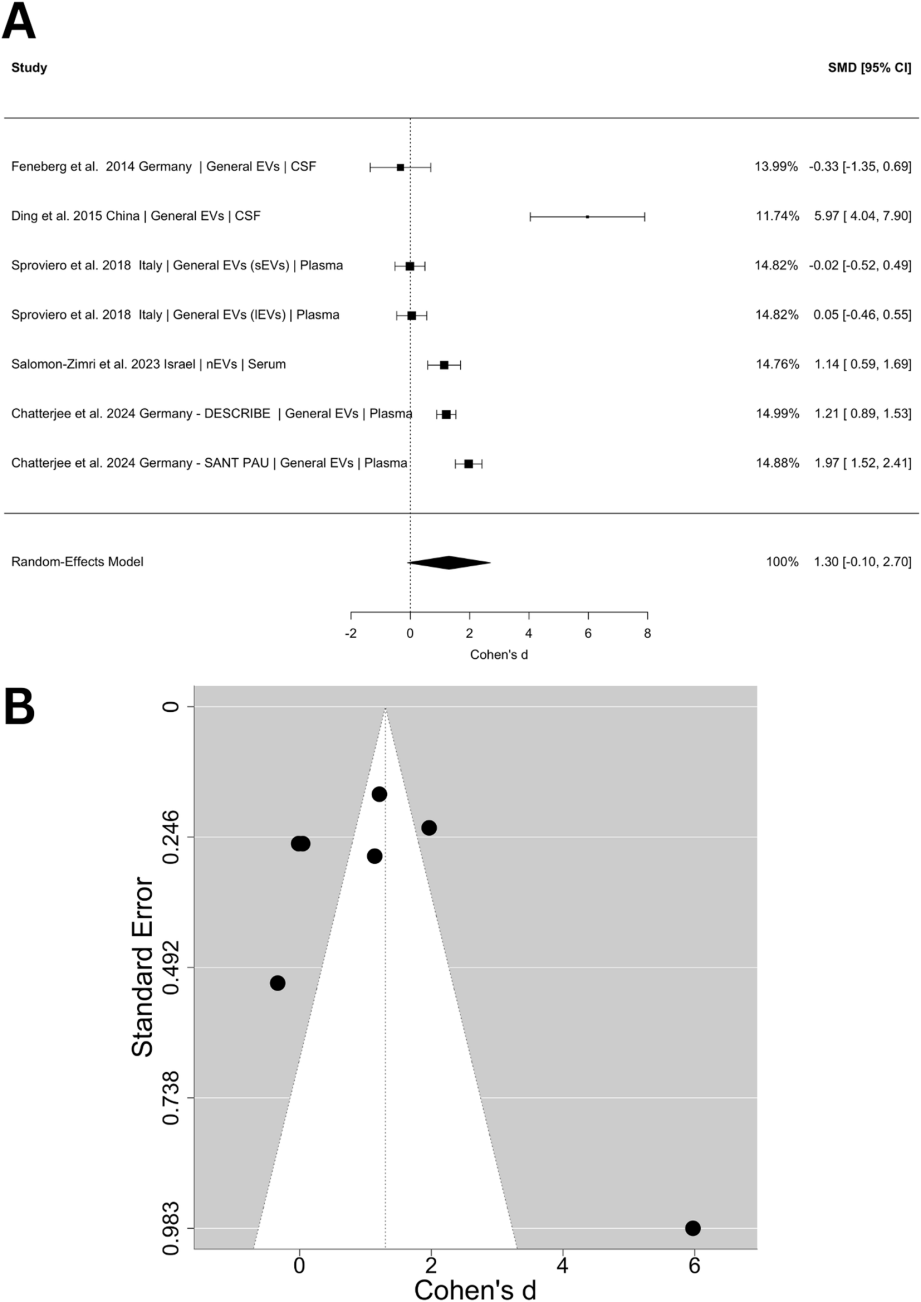
Meta-analysis of EV-associated TDP-43 levels in amyotrophic lateral sclerosis (ALS). **(A)** Forest plot summarizing standardized mean differences (SMDs; Cohen’s d) for EV-associated TDP-43 levels comparing ALS cases with controls across 5 independent studies. Study labels include country, EV subtype, and biofluid source. Squares represent individual study effect sizes scaled by study weight, with horizontal lines indicating 95% confidence intervals. The diamond represents the pooled random-effects estimate. sEVs = small extracellular vesicles; lEVs = large extracellular vesicles. **(B)** Funnel plot assessing publication bias for the included studies

The random effects meta-analysis of 5 studies [26, 28, 31, 52, 56] showed an overall increase in EV associated TDP-43 in ALS compared with controls (SMD = 1.30; Fig. 2A), though this did not reach statistical significance (*p* = 0.068), largely due to extremely high heterogeneity (I^2^ = 97.8%). One study [27] reporting low TDP-43 levels may have contributed disproportionately to this heterogeneity. We removed this outlier in a subgroup analysis, and the pooled effect increased (SMD = 1.87) and almost reached statistical significance (*p* = 0.052). A subgroup analysis excluding transformed data points did not alter the results for EV-associated TDP-43 or its borderline statistical significance, indicating that the primary finding was not driven by data transformation procedures. Importantly, additional subgroup analyses excluding duplicate contributions from the same studies (i.e., retaining only one effect size per study) did not materially change the direction or magnitude of the pooled effect. Further assessment of publication bias through Begg’s correlation (Kendall’s tau = 0.14, *p* = 0.77), Egger’s regression test (*t* = 0.28, *p* = 0.79) and visual inspection of the funnel plot suggested very minimal bias, although one study with an extreme effect size increased apparent scatter (Fig. 2B). These results suggest that more studies are needed to draw definitive conclusions on whether EV-associated TDP-43 is higher in ALS vs. controls. Another important caveat when interpreting plasma EV TDP-43 data is the abundant presence of TDP-43 in platelets, which are a potential contaminant of EV preparations [77]. None of the studies used for this random effect meta-analysis indicated the specific depletion of platelets. Without specific depletion steps or platelet-poor plasma protocols, it is difficult to exclude platelet-derived TDP-43 as a source of signal in these studies.

We did not perform subgroup analyses according to preanalytical and methodological factors, such as biofluid source, EV isolation methodology, EV characterization techniques, platelet depletion protocols, collection tube type, or EV storage conditions. Although these factors may contribute to heterogeneity and influence biomarker measurements, only five studies were available for quantitative synthesis and the majority demonstrated null findings with wide and overlapping confidence intervals.

### Other biomarkers

Beyond TDP-43 pathology, several additional biomarkers derived from EVs in ALS have been explored, reflecting diverse biological processes that extend beyond protein aggregation alone, including inflammatory and immune-related signals (IL-6, GLAST), disruptions in protein homeostasis (HSP90), markers of axonal injury (pNFH), extracellular matrix-associated proteins (PRG-4), potential endogenous retroviral activity (Retrovirus), and biophysical molecular signatures (Raman spectroscopy).

Two studies provided evidence of inflammatory changes in EVs from persons with ALS [34, 59]. IL-6 levels were reported to be higher in speculative astrocytic EVs from people with ALS compared to controls and correlated with faster disease progression [34], whereas speculative CNS-enriched GLAST positive EV counts were also increased in ALS but did not correlate with clinical severity measures, limiting their reliability [54]. Importantly, the absolute counts of leukocyte, endothelial and platelet derived EVs were similar between ALS and controls, suggesting that ALS may involve altered EV cargo rather than increased EV release from inflammatory cells [59]. Additionally, one study [45] evaluated HSP90, PPIA, and phosphorylated NFH (another marker of neuronal injury similar to pNfL) in general plasma EVs, and found that HSP90 was significantly lower in plasma-derived EVs from people with ALS compared with controls and people with spinal and bulbar muscular atrophy. This decrease was also observed in EVs from ALS mouse models, suggesting that reduced EV-associated HSP90 may represent a consistent feature of ALS pathology across both sporadic and genetic forms. In contrast, EV-associated PPIA levels did not differ among the groups, indicating limited diagnostic value, while pNFH was markedly increased in plasma from people with ALS, particularly in those with faster progression, but could not be reliably detected from EVs, suggesting that pNFH may not meaningfully be packaged into EV cargo.

Some other notable biomarkers derived from EVs have been quantified in ALS, spanning protein, viral, and spectroscopic signatures. PRG-4 was found to be significantly enriched in general EVs from persons with ALS, particularly in those with preserved cognition, and this increase was confirmed by ELISA, suggesting a possible protective or compensatory association with cognitive status [57]. A human endogenous retrovirus protein (HERV-K env), that has been discussed to be involved in the development of motor neuron disease, was measured in speculative nEVs and did not differ overall between persons with motor neuron disease (MND; 23 out of 39 with ALS) and controls, but levels were higher in individuals with classical ALS compared to other MND phenotypes and tended to increase with longer disease duration and greater lower motor neuron involvement [44]. Finally, Raman spectroscopy revealed distinct biochemical signatures in general EV populations from ALS compared to controls, with larger EVs showing increased lipid-related peaks and reduced protein-related peaks, and smaller EVs showing more subtle lipid-related shifts. Multivariate analysis of the Raman spectra modestly discriminated ALS from controls, supporting EV biochemical composition as a potential complementary biomarker of ALS [38].

### Proteomics

Twelve studies [26, 27, 37, 41, 42, 50, 57, 60, 61, 63, 65, 66] have used proteomics to evaluate biomarkers derived from EVs (see Table S2 for a summary). One study compared ALS to PD [27], one discussed EV proteomics only in relation to the TDP-43/flotillin-1 ratio [26], and the other studies compared the proteome of individuals with ALS with controls.

A proteomic study [37] using speculative CNS-enriched EVs isolated from the postmortem motor cortex of individuals with ALS identified 12 distinct proteins that were only found in ALS CNS EVs: CD177, CHMP4B, CSPG5, DYNC1I2, IGHV3-43, LBP, RPS29, S100A9, SAA1, SCAMP4, SCN2B, and SLC16A1, several of which are associated with inflammation (S100A9, SAA1, LBP), vesicle biogenesis (CHMP4B), cytoskeletal transport (DYNC1I2), or neuronal excitability (SCN2B). In addition, 16 proteins were differentially expressed between ALS and controls (VCAM1, STAU1, RRAS, PLSCR4, NT5E, ITGA5, HLA-A, GYPC, FXYD6, ENPP6, ENG, EHD1, DYNC1I1, DHX30, BST1, and AHNAK), reflecting alterations in immune signaling, RNA metabolism, membrane remodeling, and cytoskeletal function in ALS.

For completeness, 2 exploratory studies with very small n are included, although their results are not robust enough for substantive interpretation. A proteomic study that quantified CSF EVs from 3 persons with ALS and 3 persons with idiopathic normal pressure hydrocephalus identified several differentially expressed proteins, though findings are limited by the small sample size [42]. Three proteins were increased in ALS EVs (NOC2L, PDCD6IP, and VCAN), while several others were decreased, including SERPINA3, PTPRZ1, C1QC, CCDC19, MYL6B, MARCO, FCGBP, FOLR1, RELN, CFB, and CHMP4A. Another CSF EV proteomics study identified only one biomarker, bleomycin hydrolase (BLMH), which was significantly downregulated in ALS compared to controls [41]. These preliminary results point to possible EV associated changes in inflammatory, synaptic, and matrix related pathways in ALS, but none of these proteins have been reported in other proteomic studies or validated using targeted approaches. Another proteomic screen of plasma derived EVs from 3 persons with ALS and 3 controls identified twenty top candidate proteins that were markedly enriched in ALS EVs [50]. These included several RNA binding and splicing related proteins (HNRNPD, HNRNPA0, HNRNPC, HNRNPA1, RBMX, PUF60, TCERG1, SF3B3, DEK, DDX5), cytoskeletal and actin related proteins (CORO1A, ACTG1, ACTG2, ARPC1B, IQGAP1), and proteins implicated in ALS pathology such as FUS and PADI4. Many of these proteins showed more than 3-to-5-fold higher abundance in ALS EVs compared with controls, suggesting potential alterations in RNA processing, cytoskeletal regulation, and stress response pathways in circulating EVs from people with ALS. A plasma EV proteomic study that included both a discovery (12 ALS, 12 controls) and an independent validation cohort (49 ALS, 20 controls) identified 7 consistently altered proteins in ALS, all of which were related to platelet or immune pathway activity. These proteins were LBP, FGB, FGG, C9, FGA, PRG4, and VWF, highlighting a reproducible signature of coagulation and complement-associated EV cargo changes in ALS [57].

A serum EV proteomics study comparing people with ALS to controls found a distinct pattern of protein distribution rather than broad shifts across the EV proteome [61]. EVs from persons with ALS were relatively enriched in TALDO1, PTRC, PIP4K2A, LGALS7, GP9, TMP3, FGB, P4HB and CD36, whereas EVs from controls contained higher levels of SERPING1, IGHA2, IGLC3, APOC1, APOB, F11, SLC4A1 and APMAP. Among these, IGHA2 was reported to separate both groups the most. They also performed an EV metabolomics analysis, which revealed group-specific metabolic signatures. EVs from people with ALS were enriched in sphingomyelin, 5-valerolactone, 2-OH-4-methylpentanoate, 3,4-di-OH-phenylacetate, whereas EVs from controls showed higher levels of phosphatidylcholine, L-carnitine, isocitrate, suberate, deoxycarnitine, 24-hydroxycholesterol, 2-methylbutyrylglycine, and 3-methylcrotonylglycine. Another serum EV proteomics study [66] investigated differences in cryptic peptides between sporadic ALS and controls and found generally higher quantities of cryptic peptides as well as those specifically derived from IGLON5 in individuals with sporadic ALS compared to controls.

Two studies [49, 54] applied proximity extension assay to EVs in ALS but did not identify robust disease associated signals. In CSF and CSF-derived general EVs, dozens of proteins were detectable, but neither hierarchical clustering nor principal component analysis produced a clear separation between ALS and controls, and no consistently differentially expressed EV proteins emerged; only crude CSF showed partial separation driven by CHIT1 and myoglobin. The second study [54] analyzed saliva and saliva-derived EVs and also found no group separation on principal component analysis and no significantly altered proteins, with only weak trends toward lower ZNF428 in saliva EVs and higher IGLL1 in whole saliva from people with ALS. These suggest that proximity extension assays may not be a good method for finding differentially expressed biomarkers derived from EVs. Lastly, one study found increased levels of coronin-1a in plasma EVs of 3 persons with ALS compared with controls, but did not validate it using targeted-approaches in EVs [50].

Lastly, a longitudinal serum EV proteomic study [63] tracked two individuals with *SOD1* D90A ALS from their total sample (*n* = 6), isolating EVs at baseline and after one year, showing that EV protein profiles shift markedly with symptom onset and progression. Early disease was characterized by vascular and genomic stress signals, symptomatic conversion by strong immune and complement activation, and advanced disease by intensified inflammatory and phagocytic pathways. Importantly, FN1 and multiple FN1 isoforms increased consistently with clinical worsening, identifying FN1 as a potential EV-based progression marker.

## RNA

Both DNA and RNA are present in EVs and can serve as biomarkers, with EV cargo including mRNA, transfer RNA, circular RNAs and other noncoding RNAs. In our two previous meta-analyses on parkinsonian disorders [22, 23] and dementia [24], RNA-based EV biomarkers showed the highest diagnostic accuracy and overall performance compared with other biomarkers. In the context of ALS, 17 studies have quantified RNA levels derived from EVs including 6 [30, 39, 46, 51, 58, 64] using qPCR to measure specific miRNAs, 5 [33, 43, 47, 48] applying RNA sequencing or microarray approaches to identify broader panels of differentially expressed RNAs, and 6 using combined qPCR and RNA sequencing [32, 35, 36, 53, 55, 62] (see Table 1).

The main conclusion from these studies is that the findings show minimal overlap, frequently originate from the same research groups, and lack independent replication, making it difficult to draw reliable or generalizable conclusions about RNA-based EV biomarkers in ALS currently. However, it should be noted that discrepancies across miRNA studies are not solely attributable to true biological variability but may also arise from methodological differences, particularly the normalization strategies applied in semi-quantitative qPCR. Only two studies [35, 55] utilized ddPCR, an absolute quantification that can bypass qPCR normalization issues by directly estimating target copy number rather than relying on relative expression against reference miRNAs or spike-in controls. While similarities regarding pathway detection has been found across studies, only a few miRNAs were even validated via qPCRs in their own studies. This suggests that miRNAs as cargoes in EVs might be interesting molecules to study the underlying pathophysiology but need more standardization. Below we discuss a few of these studies.

One study reported the miRNA-27a-3p to be down regulated in EVs isolated from the serum of people with ALS [30]. The authors speculated that miRNA-27a-3p, a microRNA previously found to be implicated in neuronal stress responses, plays a role in mitigating the communication between muscle and bone in the context of disease, possibly reflecting dying muscle tissue in ALS. Another CSF EV study [39] reported that miRNA-124-3p, a microRNA involved in neuronal differentiation and neuroinflammation, showed a trend toward higher levels in male persons with ALS and correlated strongly (R^2^ = 0.73, *p* = 0.002) with worse disease severity (ALSFRS-R), although the group difference did not reach statistical significance. This relationship was not observed in female patients, and miRNA-124-3p levels were unrelated to age or body size, suggesting a possible sex-specific association between CSF EV miRNA-124-3p and ALS progression. A recent study analyzed plasma EV miRNAs in ALS using discovery and validation cohorts stratified by SOD1 and C9orf72 status [52]. Microarray screening followed by qPCR showed miRNA-34a-3p to be selectively reduced in SOD1-ALS and miRNA-1306-3p to be significantly downregulated in persons with genetic ALS with SOD1 or C9orf72 mutations. Both miRNA-199a-3p and miRNA-501-3p were elevated in sporadic ALS compared to controls, an effect mainly observed in males. miRNA-501-3p was further enriched in persons with bulbar-onset disease compared with spinal-onset or classical Charcot phenotypes [53], suggesting value for phenotypic discrimination. A machine-learning classifier using five miRNAs (miRNA-199a-3p, miRNA-30b-5p, miRNA-501-3p, miRNA-103a-2-5p, miRNA-181d-5p) achieved an AUC of 0.80 with 79% accuracy, supporting circulating, blood-derived EV-miRNAs as informative biomarkers that distinguish ALS from controls and capture genotype-specific signatures.

Additionally, a study using plasma EVs found altered expression of miRNA-449a and its predicted target ADAM10 in ALS compared to controls. Specifically, miRNA-449a was upregulated in EVs from persons with ALS, while ADAM10 showed an opposite, downregulated trend, supporting a reciprocal miRNA-silencing relationship consistent with neurodegenerative mechanisms [62].

Several studies assessing miRNAs in speculative CNS-enriched EVs have reported differences between ALS and controls. However, multiple studies reporting these findings [36, 51, 58] were produced by the same group, and the authors did not provide their datasets despite repeated requests. No independent group has replicated these miRNA results, and no other studies have examined these specific candidates in speculative nEVs, leaving their biomarker value uncertain. Additional studies have analyzed other biomarkers in speculative nEVs [33, 44, 52] and astrocyte-enriched EVs [34, 59], but none show overlap in identified biomarkers across groups. Importantly, speculative CNS-enriched EVs consistently show lower diagnostic performance and markedly greater publication bias compared with general EVs across multiple meta-analyses [22–24]

10 studies have examined miRNA profiles within EVs using RNA sequencing technologies [32, 33, 35, 36, 43, 47, 48, 53, 55, 62].

In one such investigation of serum-derived EVs from individuals with ALS and controls, no miRNA species reached statistical significance in association with ALS pathology [46]. The authors contrasted their findings with two prior reports [33, 36] each of which analyzed speculative nEVs isolated from plasma. These earlier studies described modest sets of differentially expressed miRNAs, 13 upregulated and 17 downregulated in one, and 5 upregulated and 3 downregulated in the other, yet no overlap emerged between them. Importantly, neither study applied correction for multiple testing, a methodological omission that substantially increases the likelihood of false-positive results [33, 36]. A recent study analyzed plasma EV-miRNAs in ALS using discovery and validation cohorts stratified by SOD1 and C9orf72 status [53]. Microarray screening followed by qPCR showed miRNA-34a-3p to be selectively reduced in SOD1-ALS and miRNA-1306-3p to be significantly downregulated in both persons with genetic ALS with both SOD1 or C9orf72 mutations, while miRNA-199a-3p and miRNA-501-3p were elevated in sporadic ALS. miRNA-501-3p was further enriched in persons with bulbar-onset disease compared with spinal-onset or classical Charcot phenotypes [78], suggesting value for phenotypic discrimination. A machine-learning classifier using five miRNAs (miRNA-199a-3p, miRNA-30b-5p, miRNA-501-3p, miRNA-103a-2-5p, miRNA-181d-5p) achieved an AUC of 0.80 with 79% accuracy, supporting circulating, blood-derived EV-miRNAs as informative biomarkers that distinguish ALS from controls and capture genotype-specific signatures. Another group focused on the analysis of mRNAs isolated from CSF-derived EVs from 4 persons with ALS and 4 controls using RNA sequencing. Along with 133 significantly upregulated and 410 significantly downregulated genes in the ALS cohort compared to controls, the group identified the ubiquitin-proteasome pathway, the oxidative stress response, and the unfolded protein response to be upregulated based on Gene Ontology analysis [32]. Of these, qPCR was used for validation of two genes (ACTB and CUEDC2) where no significant changes were detected. The ubiquitin-proteasome pathway has been also found to be enriched in plasma derived EVs from another study that investigated the miRNA signature of EVs in ALS and additional neurodegenerative studies [43].

A separate study that profiled miRNAs from plasma-derived EVs using next-generation sequencing also reported upregulation of the ubiquitin–proteasome pathway [35]. In EVs derived from persons with ALS, 5 miRNAs were upregulated whereas 22 miRNAs were downregulated compared to controls. In addition to detected changes in the ubiquitin-proteasome pathway, in silico functional annotation of predicted mRNA targets of the identified deregulated miRNAs revealed affected transcriptional regulation process. The differentially expressed miRNAs were validated using qPCR in EVs isolated from 3 controls and 12 persons with ALS. Only one of the identified significantly deregulated miRNAs was validated with ddPCR (miRNA-15a-5p) which confirmed the significant upregulation identified with next-generation sequencing. Another study profiled miRNAs in plasma-derived EVs, distinguishing between putative smaller-sized EVs and larger-sized EVs using miRNA sequencing. The analysis revealed that the miRNA profile of EVs was distinct from blood cells, indicating selective packaging of ALS-relevant miRNAs within EVs [62]. Importantly, studies employing ddPCR provide absolute miRNA quantification without the need for normalization, whereas semi-quantitative approaches such as qPCR rely heavily on normalization strategies that lack standardization in EV research. Consequently, results obtained by these fundamentally different methodologies are not directly comparable and may contribute to apparent inconsistencies across studies. In putative smaller-sized EVs 88 miRNAs were upregulated and 199 were downregulated whereas in putative larger-sized EVs 183 were upregulated and 215 were downregulated in persons with ALS compared to controls. They identified 8 miRNAs that were specifically found only in putative larger-sized EVs from persons with ALS and 22 miRNAs only in putative smaller-sized EVs from persons with ALS. While some of the differentially expressed miRNAs in putative larger-sized and smaller-sized EVs were used to perform pathway analyses, none of the identified miRNAs was validated with qPCR.

### Diagnostic accuracy

**Fig. 3:**
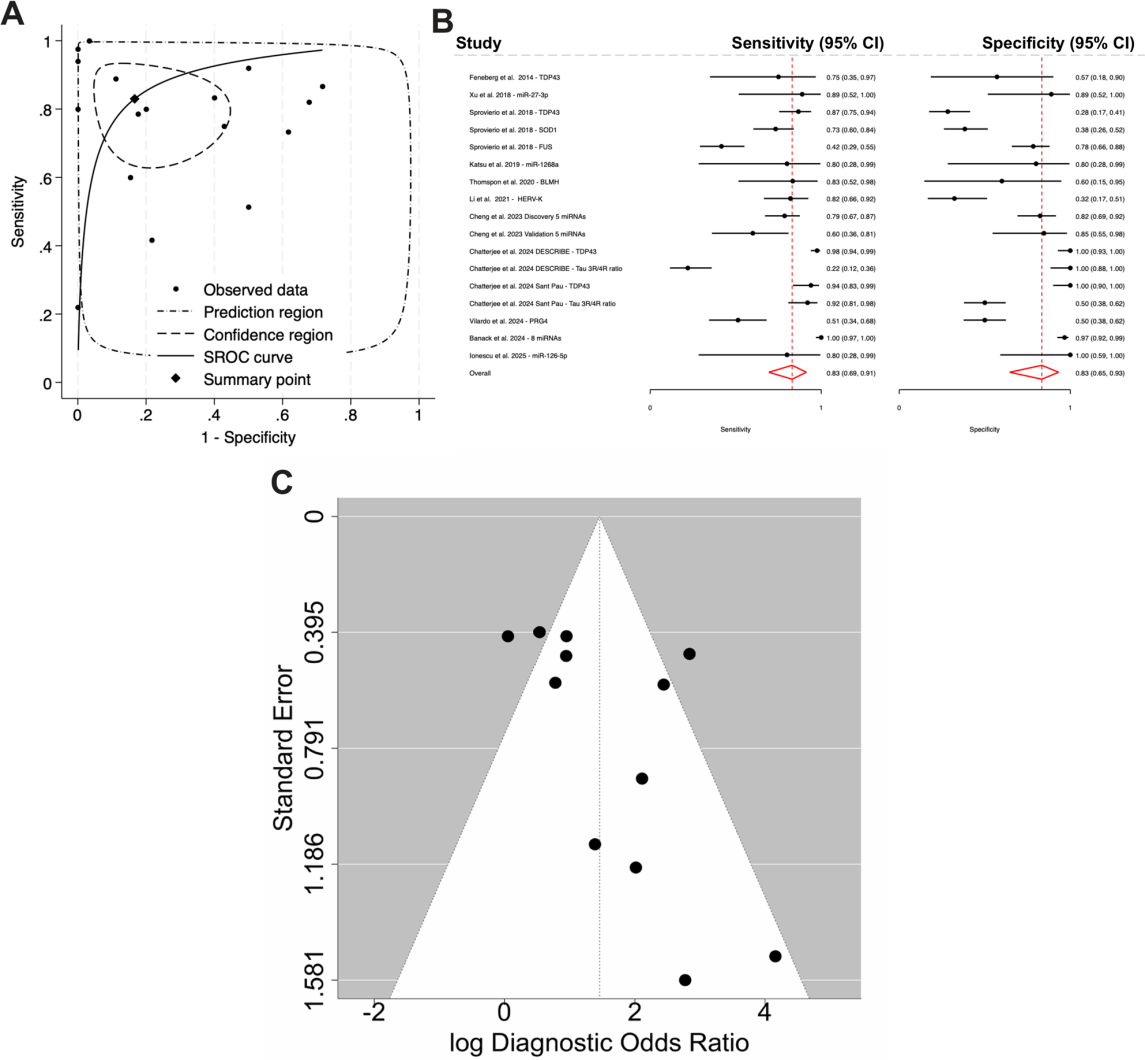
Diagnostic accuracy of general and CNS-enriched extracellular vesicle (EV) biomarkers for distinguishing ALS from controls. (**A**) Hierarchical summary receiver operating characteristic (HSROC) curve showing individual study estimates (dots), the summary point (diamond), 95% confidence region (dashed line), and 95% prediction region (dash-dot line). The solid curve represents the HSROC fit across included studies. (**B**) Forest plots of sensitivity and specificity for each biomarker study, grouped by analyte. Point estimates and 95% confidence intervals are shown for both sensitivity (left panel) and specificity (right panel), with red dashed lines indicating pooled summary estimates. (**C**) Funnel plot assessing publication bias for the included studies. Each point represents an individual study plotted by effect size (log diagnostic odds ratio) and standard error

To identify the best diagnostic biomarker(s) for ALS vs. controls, we pooled available studies using a BRMA model (Fig. 3A–B). The model revealed a moderate diagnostic accuracy (AUC = 0.839; partial AUC accounting for FDR = 0.788) and heterogeneity (I^2^ = 67.3%). As we reported previously for parkinsonian [22, 23] and dementia [24], RNA-associated biomarkers in general EVs appear to have the best combination of sensitivity and specificity (e.g., miRNA-126-5p). Publication bias assessment using Begg’s correlation (Kendall’s tau = 0.36, *p* = 0.12), Egger’s regression test (*t* = 1.82, *p* = 0.099) and visual inspection of the funnel plot (Fig. 3C) suggested no bias. When interpreting these diagnostic accuracy estimates, it should be kept in mind that sensitivity and specificity were partly derived using post hoc optimized thresholds from the same datasets, which may introduce optimism bias and means that the pooled AUC of 0.839 should be regarded as an exploratory upper-bound estimate rather than an independently validated measure of diagnostic performance.

## Discussion

In this study, we systematically reviewed the utility of EVs from 41 studies (Table 1) as a liquid biopsy for aiding in the diagnosis of ALS (Fig. 1). Out of those studies, we meta-analyzed EV-associated TDP-43 levels in 5 studies using random-effects models and 11 in the diagnostic accuracy meta-analysis. The included studies spanned a wide range of EV isolation methods, biofluids and analytical platforms. Despite this extensive literature, our findings highlight substantial methodological heterogeneity, limited replication across research groups, and inconsistent biomarker performance. Notably, the meta-analyses were based on a relatively small number of studies with considerable methodological variability, and the resulting estimates should therefore be interpreted with appropriate caution.

Across all protein focused studies, the most extensively investigated analyte was EV associated TDP-43. Although a majority of studies reported slightly higher TDP-43 levels in ALS, our pooled random effects meta-analysis demonstrated only a moderate SMD (Fig. 2A) and very high heterogeneity without reaching statistical significance. A subgroups analysis excluding one outlier study reporting unusually low values strengthened the overall effect, but heterogeneity remained high and the analysis did not reach statistical significance. Publication bias analyses using Begg correlation, Egger regression and funnel plot visualization (Fig. 2B) showed minimal asymmetry, suggesting that the observed variability mainly reflects true methodological and biological differences between studies rather than selective reporting.

RNA based biomarkers were evaluated in 17 studies, ranging from RNA sequencing to targeted qPCR analyses. In our two previous meta-analyses for parkinsonian disorders [22, 23] and dementia [24], RNA derived from general EVs consistently showed the highest diagnostic performance compared with other EV analytes. However, in ALS, the available studies displayed very poor overlap and often originated from the same research groups, which limits generalizability. But our diagnostic accuracy meta-analysis showed a moderate pooled performance with an AUC of approximately 0.84, sensitivity = 83.0% and specificity = 83.0% along with moderate heterogeneity (Fig. 3A–B) with no publication bias (Fig. 3C). However, these results remain preliminary because replication across independent cohorts is limited and validation using standardized isolation and quantification methods is lacking. Although most studies to date have relied on either protein-based or RNA-based analyses of EV cargo, single-analyte biomarkers are unlikely to capture the full biological complexity of ALS. Specifically for RNA, the 16 studies analyzed here show no meaningful overlap in reported signatures, suggesting that current EV-RNA profiles have not yet reached a level of robustness or reproducibility sufficient for clinical application. Given the multifactorial nature of the disease and the heterogeneous cellular processes reflected in EVs, future efforts will likely require integrated, multi-omics approaches [79] that combine targeted protein, RNA, lipid, and metabolite profiling. Such composite biomarker panels may offer superior diagnostic accuracy, improve biological interpretability, and better reflect the diverse pathological pathways active in ALS.

An important and currently underdeveloped application of EV biomarkers in ALS is monitoring therapeutic response. Recent biomarker studies in tofersen-treated SOD1-ALS patients have demonstrated that certain fluid biomarkers track treatment effects, highlighting the potential of therapy-responsive biomarkers to complement clinical trial endpoints. One included study [52] observed that EV-associated TDP-43 decreased with ciprofloxacin/celecoxib treatment while conventional markers remained unchanged, suggesting that EVs may capture treatment-related biological changes not reflected by standard biomarkers. Future studies should systematically evaluate EV biomarkers in the context of emerging ALS therapeutics [80–82]. Furthermore, EV-based biomarkers for ALS diagnosis should be used in a multipanel context. For example, NfL in liquid biopsies is a common and robust biomarker of neuroaxonal injury and disease activity in ALS, but it is not ALS-specific. Although elevated NfL supports the presence of an active neurodegenerative process and correlates with disease progression and survival, it has limited independent value for distinguishing ALS from other neurological disorders, particularly when used outside a clearly established clinical context [83, 84]. In this respect, EV-based biomarkers as shown in our systematic review may provide an important complementary layer of information, because EV cargo can reflect disease-relevant molecular signatures with greater biological specificity than NfL alone. Candidate EV markers such as TDP-43-associated species may therefore improve diagnostic specificity and enhance the interpretability of NfL within a multimarker framework. EV-based markers must ultimately demonstrate robust diagnostic performance, ideally with AUC values > 0.90 and sensitivity and specificity exceeding 85%. Importantly, this performance should be established against other neurological diseases rather than healthy controls alone. Future studies should also determine whether EV markers provide added value beyond established biomarkers such as NfL, using large, well-characterized cohorts and standardized methodologies to ensure reproducibility across centers.

A major source of inconsistency in the analyzed studies was the use of speculative CNS-enriched EVs. Multiple studies measuring miRNAs or proteins in neuronal or glial marker enriched EVs described differences between ALS and controls, but these studies were almost exclusively produced by the same research groups. None of the datasets were shared despite repeated requests, no independent replication exists, and the identified biomarkers had poor overlap with other studies. Moreover, prior meta-analyses have demonstrated that speculative CNS-enriched EVs perform markedly worse diagnostically and exhibit substantially greater publication bias compared with general EVs [22–24]. This is further compounded by ongoing uncertainty about their biological origin since the markers used for enrichment are not specific to neurons, exist in soluble form and EVs originating from various tissues undergo extensive circulation and uptake which likely dilutes any original signal from the CNS. An additional emerging concern is the biomolecular corona, a layer of proteins that adsorbs onto EV surfaces during isolation and may be misidentified as genuine EV cargo unless rigorously characterized, for example by electron microscopy. None of the included studies systematically addressed this issue, which may have contributed to variability in reported cargo profiles. Collectively, these factors mirror our previous meta-analyses showing that speculative CNS-enriched EV-associated biomarkers are unreliable for parkinsonian disorders [22, 23] and dementia [24], and raise significant concerns regarding their reliability, reproducibility, and validity as clinical biomarkers. Under certain circumstances, internally validated speculative CNS-enriched EV assays may still show promise, but concerns remain about the cross-study comparability. Standardization efforts should align with the guidelines set by the International Society for Extracellular Vesicles (ISEV), whose Minimal Information for Studies of Extracellular Vesicles (MISEV) position papers provide consensus recommendations for EV isolation, characterization, and reporting [85].

### Limitations

Several limitations of this study warrant consideration. First, the meta-analysis was conducted on a small number of studies with substantial methodological heterogeneity, including differences in biofluid source (plasma, serum, defibrinated plasma, CSF), EV isolation method (ultracentrifugation, polymer-based precipitation, membrane affinity), and quantification platform (mass spectrometry, ELISA, single molecule array, qPCR). This heterogeneity likely underlies the high I^2^ values observed and limits the interpretability of pooled estimates. Second, for studies that did not report sensitivity and specificity at a predefined diagnostic threshold, these metrics were derived post hoc by conducting binomial logistic regression on extracted or reconstructed data and selecting the threshold maximizing Youden’s index. This constitutes re-analysis rather than extraction of reported diagnostic performance and is subject to optimism bias, particularly in small cohorts without internal resampling or external validation. Reported sensitivity and specificity values should therefore be interpreted as model- and threshold-dependent estimates rather than validated diagnostic performance. Third, for one study, medians and interquartile ranges were converted to means and standard deviations using an established formula; this conversion assumes approximate normality of the underlying distribution, which may not hold for biomarker data that are frequently right skewed. Fourth, several ROC analyses included in the diagnostic accuracy meta-analysis were conducted on small datasets, which increases the risk of overfitting and may inflate apparent diagnostic performance. Fifth, an emerging methodological concern across the EV field is the biomolecular corona, a layer of proteins that adsorbs onto EV surfaces during isolation and may be misattributed as genuine EV cargo in the absence of rigorous characterization such as electron microscopy. None of the included studies systematically addressed this, which may have contributed to variability in reported cargo profiles. Sixth, platelet depletion was rarely documented, with only 7 of the 30 studies explicitly reporting platelet-free sample preparation. This is a major limitation for TDP-43 analyses because platelets are an abundant source of circulating TDP-43 and may co-isolate with EVs, and thus a contribution of platelet-derived TDP-43 to the observed signal cannot be ruled out [86]. Seventh, several research groups declined to share underlying datasets despite repeated requests, precluding inclusion of those studies in the meta-analyses and introducing a potential source of selection bias.

## Conclusion

Overall, the present synthesis highlights both the promise and the limitations of EV based biomarkers in ALS. General EVs appear more robust and reproducible than speculative CNS-enriched preparations and protein biomarkers such as TDP-43 show biologically consistent but methodologically variable results. RNA signatures remain appealing due to their diagnostic performance in other neurodegenerative conditions, but ALS specific studies require significantly more replication and methodological standardization. Future investigations should prioritize transparent data sharing, harmonized EV isolation protocols, rigorous validation using orthogonal assays and replication across independent cohorts.

## Electronic supplementary material

Below is the link to the electronic supplementary material.

Supplementary Material 1

## Data Availability

All data analyzed are included in the article.
